# A Case Presenting Choreic Symptoms, Terminating in Death

**Published:** 1858-09

**Authors:** O. C. Gibbs

**Affiliations:** Frewsburg, New York


### Original Communications.

ART. I.—*A Case Presenting Choreic Symptoms, Terminating in Death.* By O. C. GIBBS, M.D., of Frewsburg, New York. Read before the Medical Society of Southwestern New York, February 3d, 1858.

CHOREA, uncomplicated with other diseases, is seldom fatal, and, in the onset of the attack, is usually amenable to treatment. The case which I am about to relate possesses some points of interest; and, though wanting the completeness of post-mortem observation, may not be wholly unworthy of record.

On the 15th of June, 1857, I was called to see Miss Cowen, aged thirteen years, and found her laboring under severe local pains, affecting principally, though not exclusively, the lower extremities. The pains, which were paroxysmal and migratory in their character, attacked most frequently some of the joints of the extremities; but occasionally seemed to be located in the muscular tissue; sometimes in the head, side, etc. There was no swelling or redness of any of the parts affected. The tongue was slightly coated with a whitish fur, and the pulse was accelerated to a hundred beats a minute. She was sweating profusely and continuously; the sweat standing in great drops upon the surface of the body, and not unfrequently dropping from the nose, cheeks, and other parts of the face.

She had been suffering from these symptoms for about a week, previous to which time her health had been pretty good. She had never menstruated, nor had she seemed to suffer from a want of establishment of this function. At the time of my visit she was sitting up, was cheerful, and in good spirits.

The absence of the catamenia, the age and rather slender constitution of the patient, excited suspicions of hysteria; but, in view of all of the circumstances, I could not look upon the case as one of the protean manifestations of that disease. Suspecting that the symptoms had a rheumatic origin, I ordered five grains of calomel to be taken immediately; to be followed, in four hours, by black draught sufficient to procure free catharsis. Five grains of Dover's powder and twenty drops of the wine of colchicum were ordered to be given every six hours, to be commenced with immediately after the operation of the cathartic.

Under this treatment the local pains soon disappeared, and the pulse became less frequent; the sweating, however, continued profuse, and the anæmic condition of the patient became more marked. On the fourth day of treatment I discovered some irregular muscular motion, apparently of a choreic character, about the shoulder, arm, and hand of the right side. There was an almost incessant shrugging of the shoulder and working of the fingers. When voluntary motion was attempted with the affected hand it was performed with unsteadiness and irregularity, but always successfully. I now ordered the tincture of cimicifuga, in drachm doses, and the tincture of chloride of iron, in doses of ten drops, each to be repeated every six hours. This treatment was continued for three days; the choreic symptoms gradually increasing. The deficient nervous integrity extended to the leg of the affected side; walking was accomplished with difficulty, and the foot seemed to drag after the other. The patient wept often, and without apparent cause, and smiles and tears were not unfrequently present at the same time. As the tincture of chloride of iron did not seem to set well upon the stomach, powders, composed of one grain of sulphate of quinine and five of subcarbonate of iron, were substituted; the tincture of cimicifuga being administered as before. The bowels were kept regular with pills composed of aloes and blue mass. Local pains and restlessness were quieted with morphine. It may be well to remark here, that the last two items of direction were left, for three weeks, to the discretion of friends, and the laxative and the anodyne were given as the necessities of the case seemed to require. This treatment was continued for four or five days; the patient seeming to improve under its employment. I have neglected to state that the profuse sweating had continued up to this time without abatement. Sweating seemed more profuse during the night than throughout the day; but she sat up much during the day, while in the night evaporation was prevented by the bedclothes, and the difference was probably more seeming than real. The cimicifuga was still continued, and for the powders of quinine and iron, pills, composed each of one grain of sulphate of zinc and four of extract of hyoscyamus, were substituted. Zinc has, for some time, enjoyed no inconsiderable reputation in the treatment of chorea; and Dr. Theophilus Thompson, of London, in his "Clinical Lectures on Pulmonary Consumption," Dr. George H. Barlow, also of London, in his "Manual of the Practice of Medicine," and Dr. E. P. Coxe, Physician to the Charity Hospital, New Orleans, in a paper published in the *Boston Med. and Surg. Journ.*, April 8th, 1857, have strongly recommended a combination of zinc and hyoscyamus in the profuse night-sweats occurring in the last stages of consumption. Hence, it was with the hope of accomplishing a double purpose that zinc was ordered as above. This treatment was continued for five or six days; the patient continuing to improve. The choreic symptoms had nearly passed away, the strength and appetite had improved, and the patient was able to ride out, and also to walk about the yard. Simultaneous with the gradual improvement in the choreic symptoms, the paroxysmal local pains returned, and, although the general health was considerably improved, yet there was but slight decrease in the profuse sweating, and the local pains were nearly as severe as at first.

I now ordered two grains of strychnia in two ounces of water, with the addition of a few drops of diluted sulphuric acid. Twenty drops of this solution to be given every six hours; the use of the wine of colchicum and the Dover's powder, mentioned above, to be continued. This treatment was kept up for four or five days, at which time the local pains had nearly passed away, but the choreic symptoms had returned as severe as at any previous time. The same side as before was the seat of irregular muscular action; but now, in addition to the choreic symptoms, there seemed to be a partial hemiplegia. The face, on the side affected, was almost devoid of expression, and, in laughing, the mouth was drawn to the opposite side. I told her to grasp my finger with the affected hand, and in doing so she carried her hand to mine with that peculiar zig-zag motion so characteristic of chorea, but when once she had hold of my finger she grasped it with firmness. In view of this fact, which was deemed all-important, inasmuch as the mind was cheerful, although the patient was occasionally subject to paroxysms of weeping, and in view also of the well-known fact that partial palsy is not infrequent in chorea, I did not diagnose serious lesion of the brain. I now returned to the tincture of cimicifuga, the pills of zinc and extract of hyoscyamus, and, in addition, gave the ferrocyanuret of iron, in five-grain doses, every six hours.

This treatment was continued for four days, with some abatement of the choreic symptoms, though the partial paralytic condition remained unimproved. She walked about the house and the garden, leaning upon a friend; her appetite was quite good, and she rode out several times. At this time the tongue became suddenly and completely palsied; she could not project it, and all power of speech was lost; she seemed in other respects not worse; she would laugh at her own futile attempts at conversation, and would as often weep over her unfortunate affliction. As it was the right side which was affected, she could not converse with her friends in writing, and hence amused herself and took great pleasure in pointing to letters in the alphabet, forming words and sentences, thus expressing her thoughts and making known her wants and feelings.

The sweating had continued, with but little diminution, from the onset; the head was neither hot nor painful; there was no intolerance of light; the pulse was never hard nor irregular, and up to this time it had never fallen below ninety, nor risen above a hundred beats. During the progress of the case, the symptoms were not unlike such as might have arisen from chorea, complicated with hysteria; but having, perhaps, an unwarrantable confidence in my ability to diagnose the multiform manifestations of that disease, I could not avoid taking a more serious view of this case. The true pathological significance of the symptoms, however, to my understanding, was veiled with more or less of mystery, and encompassed with doubt.

Having but little confidence in the therapeutic powers of the ferrocyanuret of iron, it was discontinued, and, in view of the paralytic symptoms, the solution of strychnia was substituted; the other treatment remaining the same. This treatment was kept up, and the case progressed without much change for the next four days, when I was hastily summoned to the patient, and, on my arrival, found her in a state of coma or insensibility, from which she could not be aroused. The countenance, lips, and surface generally, were of a snowy whiteness; the pulse was more frequent, being 120, and was very feeble, but quite regular. The sweating, however, was not so copious; the breathing was slightly accelerated, but not labored. Liquids placed in the mouth were slowly swallowed, apparently without consciousness or difficulty. The pupils were not materially contracted nor dilated.

What was the cause of the coma, was a question not altogether easy of satisfactory solution. Had meningeal, or local cerebral inflammation been slowly and unobservedly progressing to a state of effusion? In view of all the symptoms, I was not fully prepared so to decide. Was the coma not rather owing to an insufficient supply, or rather to a supply of imperfect blood to the brain, resulting in a depression of the cerebral functions? I

was rather inclined to the latter opinion; but must confess I was not firm in the conviction of its correctness, and, consequently, did not clearly see the indications of treatment.

As the bowels had not acted for two days, I gave five grains of calomel, and one-fourth of a grain of podophyllum, rubbed up with a little sugar. This combination was chosen partly on the account of the smallness of the dose, and because it could be dropped upon the tongue, from whence it could be readily washed down with any simple drink. A blister, two inches in width and ten inches in length, was applied over the cervical and upper portion of the dorsal vertebræ, and another of similar dimensions was applied across the chest in the infra-clavicular region. Watching the case closely for an hour, I finally concluded to pursue, for a time at least, a stimulating and tonic course. Be the pathological condition which gave rise to the cerebral symptoms what it might, it seemed evident, in view of all the symptoms, that death, which appeared imminent, could best be everted, at least temporarily, by a stimulating plan. Hence carbonate of ammonia and quinine, in diluted brandy, were ordered. On the following morning my friend, Dr. Axtell, saw the case with me. At this time there was more color to the countenance, and more force and fullness to the pulse. With much effort the patient could be aroused so as, apparently, to have a vague conception of what was said to her. The bowels had moved two or three times from the cathartic given the day previous. Dr. Axtell was of opinion that effusion, to some extent, had taken place in the cranial cavity, and hence that stimulants were contraindicated. The iodide of potassium, in two-grain doses, was ordered to be given every four hours, and, at the suggestion of Dr. Axtell, a small powder of hydrargyrum cum creta was ordered every eight hours, and a blister applied over the anterior surface of the neck. Without manifesting any improvement, the patient lingered for ten days longer, when she died. I have no minute record of the last ten days' treatment; suffice it to say, however, that the iodide of potassium and the counter-irritation were continued until death. From the onset of the coma until death, there was no irregular muscular action, either of a choreic or convulsive character. An opportunity for an autopsy was earnestly solicited, but was strenuously declined. After the patient's death, I learned one fact that may have had some connection with the origin of the disease. About two weeks previous to the commencement of the illness, she jumped from a fence, striking erect, and upon her feet. She immediately complained of having been hurt by the jump, and referred the seat of injury to the back of the neck. For four or five days she carried her head to one side, and suffered from pain in her head and back; but for ten days subsequently she was apparently well, or at least did not complain.

Was this a case of meningeal or local cerebral inflammation terminating in effusion upon, or within the brain? or was it, rather, a case of chorea occurring in a delicate female, terminating in death by anæmia or by inanition, and probably without apparent organic lesion of the brain? I confess to an inclination to the latter opinion, and although, in the absence of an autopsy, all opinions must be conjectural, I subjoin a few ideas in support of that opinion.

In the first place, the profuse sweating, with a cool skin, and without feverish excitement, was not indicative of inflammation; if not connected with anæmia as a cause, it certainly rapidly tended to that condition as an effect. The local pains were unaccompanied with heat, redness, or swelling, and, consequently, were not directly, and probably not indirectly, connected with an inflammatory condition.* The general cheerfulness, unattended with mental exaltation or depression, the regular pulse and natural pupil of the eye, were not symptoms generally present in inflammation affecting the brain or any of its appendages. The irregular muscular action was evidently choreic, and the coma, conjoined with a regular, but feeble pulse and a natural pupil, without any convulsive manifestations, did not argue strongly in favor of effusion.

A few thoughts in regard to the nature of chorea will, perhaps, place this case in a stronger light, and will not inappropriately conclude this paper. Many investigators have sought diligently for the lesion which determines choreic convulsions; but as death seldom takes place from the uncomplicated disease, the researches of pathological anatomists have been made under unfavorable circumstances. The cerebrum, cerebellum, and spinal cord have been repeatedly and thoroughly examined for the causative lesions upon which chorea depends Various changes have been observed, but so diversified in their character, and so manifestly connected with complications, as to justify the conclusion that they were unconnected with choreic symptoms. Hence the researches of the pathologist have thrown no light upon the nature of the disease under consideration. Physiologists have described a causative irritation, which, from its nature, must, perhaps, forever escape the vision of the anatomist; they have attempted to locate that irritation, but so far, I believe, without uniformity of result. The pathology of chorea has been the subject of much controversy; yet it is admitted by all that the primary irritation is in the nervous system; but whether in the brain or medulla spinalis, is a question upon which the controversy hinges. The late Dr. Marshall Hall was of

* This probability is based upon the supposition that local pains may occur in a rheumatic diathesis—pains which we call rheumatic—unaccompanied with rheumatic inflammation or fever.

the opinion that the primary irritation was seated in the spinal marrow; Dr. Robert B. Todd and others refer it to the brain itself. In the absence of post-mortem evidence, an opinion, in reference to the seat of the primary irritation, must be based upon the physiology of the nervous system, as productive of muscular motion, whether connected with or independent of volition. All voluntary movements, all acts of volition and sensation, receive their stimulus from the brain, and cannot take place without the agency of that organ. The involuntary movements do not of necessity involve sensation, and are performed without any participation of the will,—it may be in opposition to it; they are called forth by stimuli applied to the peripheral extremities of the afferent nerves, and reflected through the spinal cord.

Dr. Todd, in his "Lumleian Lectures" on the pathology and treatment of convulsive diseases, published in the *Medical Gazette*, 1849, says:—"The brain and spinal cord (and I beg to state that I use these terms to signify, respectively, the intra-cranial and intra-spinal nervous mass,) as the great centres of the nervous system—the great nervous battery of the body—show distinctly a division into two portions: one in which nerves are implanted; the other, which has no immediate connection with nerves, and communicates with them only through the medium of the former part." (Lecture II. p. 726.) Now, if I rightly understand Dr. Todd, in the extract given above, and we restrict the brain proper to that portion of the intra-cranial mass which has no connection with nerves, I can hardly see how the primary irritation in chorea can be referred to the brain itself. With Dr. Todd's views on the physiology of the nervous system and of muscular motion, which I have not the space here to illustrate, the arguments of Dr. Carpenter and others, in proof of the purely cerebral origin of chorea—namely, the peculiar connection of the disordered movements with emotional excitement, and their cessation during sleep—seem to me devoid of force. The jactitation and involuntary character of the convulsive movements, performed in opposition to the will, and but limitedly under its control, point to some portion of the automatic apparatus as the seat of the primary lesions in choreic convulsions. An able writer thus contradistinguishes the automative and cerebral actions:—"The sensorial ganglia, medulla oblongata, and spinal cord are the immediate instruments of all sensorial and motor changes; by their sole and independent action are produced all those movements which, being unprompted by the will, or even taking place in opposition to it, are said to be automatic. These automatic movements may be excited either by sensation or by impressions which do not necessarily affect the consciousness."

"On the other hand, the cerebrum is the instrument of purely mental operations, and has no direct connection with the external world. It receives all its stimuli to action through the sensorial ganglia; and it exerts all its influence upon the muscular system through the centres of automatic movement. Hence it may be said to be played upon by the sensorial portion of the automatic apparatus, and to play upon its motor portion. An *idea* with which a pleasurable or painful *feeling* is associated, may act emotionally (as we say) upon the automatic apparatus, without any voluntary direction, or even in opposition to it. The will may be exerted in antagonizing, strengthening, guiding, or controlling the automatic actions; but, in every case, the muscle directly receives its power from the automatic centre; and the very same movement may be automatic, emotional, or voluntary, according as the motor influence has been excited in the centre from which the nerve proceeded, by an external stimulus acting through an afferent nerve, or by an emotional impulse transmitted downward from the sensorial centres, or by a volition originating in the cerebrum." (*British and Foreign Medico-Chirurgical Review*, vol. v. pp. 17 and 18.) Admitting this view of the cerebral and automatic actions, and drawing the distinctions, as above, between the brain proper and the automatic apparatus, the chain of sensora ganglia, at the base of the brain, can only be regarded as the compliment of the spinal cord, in the formation of the automatic centre on which the cerebrum is superpoised.

Having, then, established the fact that choreic convulsions are excited by irritation, primarily within the automatic centre, or reflected to it from the peripheral extremities of afferent nerves, the next inquiry is, whether that irritation is seated within the spinal cord, medulla oblongata, or sensorial ganglia.

Choreic convulsions are occasionally followed by partial paralysis and hemiplegia; from this fact Dr. Todd argues that the seat of the irritation is intra-cranial, and above the decussation of the anterior pyramids. He locates the primary irritation in that portion of the *sensorial* ganglia which is most nearly connected with the plantation of the auditory and optic nerves. This opinion very nearly harmonizes with that expressed by Dr. Carpenter; and, though located within this complimental portion of the spinal cord, this topmost portion of the automatic centre,—the sensorial ganglia,—it is not within the spinal cord itself, as supposed by Dr. Marshall Hall. If additional arguments are wanting, aside from the one given, namely, the frequency of choreic hemiplegia, to prove that the primary irritation is not in the spinal cord, I may mention the frequent origination of chorea from mental causes, and also the cessation of the characteristic jactitatory movements under the influence of sleep. That the seat of irritation is not in the brain proper, derives additional confirmation from the fact that the consciousness is not affected even in the severest forms of the complaint. It may be thought that the case adduced militates against this last assertion; but it should be remembered that the coma was evidently the result of complications, and not the legitimate effect of choreic causative irritation.

Although physiologists have determined the seat of the primary irritation with a good degree of probability, yet we have no reason to expect that the pathological anatomist will ever find there, as a choreic cause, any serious organic lesions. In fact, it is next to certain that no such lesions exist. In tetanus, whether resulting from its own natural poison, or artificially induced by strychnine, there is a greater disturbance of the automatic centres than in chorea. In tetanus, unlike chorea, deaths frequently occur uncomplicated by other diseases. Yet in death resulting from tetanus, pathologists have so far failed to detect any evidences of organic lesion in the cerebro-spinal centres, and we have reasons for concluding that, if the severe tonic spasms of tetanus are independent of organic lesion, the milder jactitations of chorea are no less so.

What is the nature of this irritation, seated probably in some portion of the sensorial ganglia, and causative of chorea? It is said this affection may be produced by intestinal irritation, such as worms, constipation, vitiated secretions, etc.; by fright, cold, etc.; but is it not probable that a choreic idiosyncrasy or diathesis must previously exist?

Cold and catarrh are numbered among the causes of phthisis, but a tubercular diathesis must previously exist; the local disease is the result of a constitutional cause, involving the nutritive function, and stamping that function with its peculiar idiosyncrasy. Gout may succeed a debauch, but without the pre-existence of a gouty diathesis, tainting the circulatory system with its poisonous products, the debauch would prove inert, so far as the development of the painful local symptoms is concerned. Though choreic convulsions result from an irritation in the sensorial portion of the automatic apparatus, yet it is highly probable that this irritation is preceded by a constitutional affection—a choreic diathesis in some way involving the blood, resulting in depraved nutrition, and in a weakened and irritable condition of the automatic centres. Dr. Todd supports this view of the nature of chorea: upon this point he says "the disease is essentially one of depraved general nutrition." Dr. Simon says "chorea confines its attacks to individuals in whom the blood development is obviously defective."* (*Simon's Pathology*, p. 154.) Not only in chorea, but in all diseases where exaltation of function is evinced by the cerebro-spinal centres, as a primary nervous phenomenon, it is highly probable that the

* See a paper by the author in the Medical Examiner for 1856, p. 525.

irritation in the automatic apparatus is caused by some influence of which the blood is the vehicle.

To bring these remarks to bear upon the case reported, I would say that it is highly probable that the patient inherited, or had acquired, a choreic diathesis, which was ready to be developed into activity, on the application of any cause capable of acting directly, or by transmission, upon the sensorial ganglia. This idea derives some support from the fact that the patient once before had chorea, which continued for two or three months, and then subsided under treatment. The more immediate causes of the present attack were—first, an anæmic or chlorotic condition, giving rise to a weakened nutrition of the cerebro-spinal centres, with some degree of irritation which, Dr. Todd says, is the true nature of the cerebral affection in chorea; and, second, the injury done, and irritation developed at, or conveyed to the base of the brain, or rather, probably, to the sensorial ganglia, by the jump from the fence, as previously mentioned. As to the local pains, it is only necessary to remark that it has been thought by some that the choreic idiosyncrasy is identical with, or similar to the rheumatic diathesis. I believe that Drs. Addison and Todd favor this idea. I am not ignorant of the fact that Dr. Simon objects, but that objection is worthless, when we remember that he admits that a rheumatic diathesis is "capable of producing that irritation of the nervous centres which is characteristic of chorea." Neither am I ignorant of the fact that Dr. Simon says that "rheumatism and chorea are never coincident in their occurrence." Upon this point I wish simply to say that I do not desire to be understood as asserting that the local pains *were* positively rheumatic. The immense sudorous evacuations which accompanied the case, it is true, incline me to the opinion that such was their nature; yet every one knows that severe local pains and exhausting fluxes are no infrequent accompaniments of anæmia.

The colliquative diaphoresis, which continued in spite of treatment, served but to perpetuate and aggravate all the symptoms that had an anæmic cause.

The coma may have been the result of a depression of the cerebral functions, or effusion may have taken place upon the brain, or within its ventricles; but, if coma was the result of effusion, it is highly probable that that effusion was not the result of inflammation, but of an impoverished and watery condition of the blood. It is a well-known fact that flux and dropsy may result from anæmia as well as from plethora; from asthenic as well as from sthenic hyperæmia.

